# Lipopolysaccharide induces neuroinflammation in microglia by activating the MTOR pathway and downregulating Vps34 to inhibit autophagosome formation

**DOI:** 10.1186/s12974-019-1644-8

**Published:** 2020-01-11

**Authors:** Xiaoxia Ye, Mingming Zhu, Xiaohang Che, Huiyang Wang, Xing-Jie Liang, Chunfu Wu, Xue Xue, Jingyu Yang

**Affiliations:** 1 Department of Pharmacology, Shenyang Pharmaceutical University, Shenyang, 110016 People’s Republic of China; 2 CAS Center for Excellence in Nanoscience, CAS Key Laboratory for Biomedical Effects of Nanomaterials and Nanosafety, National Center for Nanoscience and Technology of China, Beijing, People’s Republic of China; 3 State Key Laboratory of Medicinal Chemical Biology and College of Pharmacy, Nankai University, Tianjin, 300071 People’s Republic of China

**Keywords:** Lipopolysaccharide, Microglia, Autophagy, MTOR, Vps34, Neuroinflammation

## Abstract

**Background:**

Microglial activation is a prominent feature of neuroinflammation, which is present in almost all neurodegenerative diseases. While an initial inflammatory response mediated by microglia is considered to be protective, excessive pro-inflammatory response of microglia contributes to the pathogenesis of neurodegeneration. Although autophagy is involved in the suppression of inflammation, its role and mechanism in microglia are unclear.

**Methods:**

In the present study, we studied the mechanism by which lipopolysaccharide (LPS) affects microglial autophagy and the effects of autophagy on the production of pro-inflammatory factors in microglial cells by western blotting, immunocytochemistry, transfection, transmission electron microscopy (TEM), and real-time PCR. In a mouse model of neuroinflammation, generated by intraventricular injection of LPS (5 μg/animal), we induced autophagy by rapamycin injection and investigated the effects of enhanced autophagy on microglial activation by enzyme-linked immunosorbent assay (ELISA) and immunohistochemistry.

**Results:**

We found that autophagic flux was suppressed in LPS-stimulated N9 microglial cells, as evidenced by decreased expression of the autophagy marker LC3-II (lipidated form of MAP1LC3), as well as increased levels of the autophagy adaptor protein SQSTM1. LPS significantly decreased Vps34 expression in N9 microglial cells by activating the PI3KI/AKT/MTOR pathway without affecting the levels of lysosome-associated proteins and enzymes. More importantly, overexpression of Vps34 significantly enhanced the autophagic flux and decreased the accumulation of SQSTM1 in LPS-stimulated N9 microglial cells. Moreover, our results revealed that an LPS-induced reduction in the level of Vps34 prevented the maturation of omegasomes to phagophores. Furthermore, LPS-induced neuroinflammation was significantly ameliorated by treatment with the autophagy inducer rapamycin both in vitro and in vivo.

**Conclusions:**

These data reveal that LPS-induced neuroinflammation in N9 microglial cells is associated with the inhibition of autophagic flux through the activation of the PI3KI/AKT/MTOR pathway, while enhanced microglial autophagy downregulates LPS-induced neuroinflammation. Thus, this study suggests that promoting the early stages of autophagy might be a potential therapeutic approach for neuroinflammation-associated diseases.

## Introduction

Microglia, the resident macrophage population in the central nervous system (CNS) [1], respond to subtle changes in their local microenvironment and maintain homeostasis in the brain. Under physiological conditions, microglia play an important role in neuronal survival by producing neurotrophic factors as well as by phagocytosing dead cells, protein aggregates, cellular debris, and invading pathogens [2]. Accordingly, reduced phagocytotic activity of microglia may result in AD-associated neuroinflammation [3–7]. Microglia respond quickly to various stimuli. Following stimulation, microglia undergo morphological changes in morphology and proliferation, secreting a variety of cytokines, chemokines, and neurotoxic factors, which in turn, play critical roles in regulating neuronal activity and function [8]. Preclinical and clinical research has proved that excessive activation of microglia triggers the release of diverse pro-inflammatory factors such as reactive oxygen species (ROS), tumor necrosis factor α (TNF-α), interleukin-6 (IL-6), interleukin-1β (IL-1β), and nitric oxide (NO), which are believed to result in neuronal insult as well as aggravating the progression of neurodegenerative diseases [1, 9–13]. Currently, molecular profiling at single-cell resolution during the process of neurodegeneration is providing a new opportunity to better understand how immune cells in the CNS respond during inflammation [14]. In a recent study, armed with the single-cell data for each of the relevant cell type, Lin et al. revealed that APOE4 microglia-like cells exhibited altered morphology which was correlated with enhanced inflammatory transcriptomes and impaired uptake of Aβ1-42 in a stem cell–based model of Alzheimer’s disease [15]. Additionally, alleviating the excessive pro-inflammatory response of microglial cells by pharmacological or genetic methods has been identified as a novel therapeutic approach to manage neurodegenerative diseases.

Autophagy is an evolutionarily conserved and lysosome-dependent protein degradation process that maintains cellular homeostasis. Autophagy depends on vesicles called autophagosomes, which are formed from crescent-shaped membrane structures called phagophores or isolation membranes. Phagophores are derived from ER subdomains known as omegasomes. Vps34 is a class III phosphoinositide 3-kinase (PI3KIII) that generates phosphatidylinositol 3-phosphate (PtdIns3P, PI(3)P) on the ER, which recruits the PI(3)P-binding protein DFCP1 (double FYVE-containing protein 1) and promotes omegasome formation. Maturation of the omegasomes to phagophores involves the PI(3)P effector protein WIPI2 (WD-repeat domain, phosphoinositide interacting) [16–18]. Recruitment of Atg proteins and lipid conjugation of LC3 are required for phagophore expansion and autophagosomal membrane formation. The autophagosomes are eventually transported to lysosomes for degradation of their sequestrated contents [19–21]. Autophagy has a major cytoprotective effect in neurons, since defective autophagy or mitophagy (autophagic clearance of mitochondria) is frequently observed in association with neuronal loss and cognitive decline in aging or in CNS neurodegeneration [22]. A failure to eliminate neurotoxic proteins, as a result of impaired autophagy/mitophagy, causes spontaneous neurodegeneration, while restoration of autophagy/mitophagy is neuroprotective in Alzheimer’s disease [23, 24].

Autophagic dysfunction in neuronal or glial cells is widely associated with neurodegenerative diseases [25–27]. Microglial activation is strongly linked to neuroinflammation, and more and more evidence shows that autophagy within microglia is involved in the regulation of neuroinflammation and neurobehavior [28]. Su et al*.* demonstrated that autophagy inhibition participates in the excessive pro-inflammatory response of brain macrophages or microglia and autophagy controls the inflammatory response in microglia [29, 30]. Moreover, Ji et al. reported that the enhancement of autophagic activity facilitates the M1-to-M2 shift of microglia [31]. Although proper activation of microglia can be beneficial for microenvironment reconstruction, excessive pro-inflammatory response of microglia will aggravate the damage. Thus, correcting the dysregulation of autophagy and reducing the dysfunction of microglial cells have been proposed as potential therapeutic approaches to treat neurodegenerative diseases. However, the relationship between microglia and autophagy and the underlying mechanism by which autophagy regulates microglial inflammation are not well understood.

Here, we provide evidence that the autophagic process in microglia is impaired by LPS activation, and this occurs through suppression of autophagosome formation rather than through a change in the function of lysosomes. Moreover, a significant alleviation of inflammation was observed after the activation of autophagy by rapamycin. Therefore, the present study indicates that promoting autophagy at the stage of autophagosome biogenesis may be a novel therapeutic approach to treat neuroinflammation.

## Methods

### Reagents

Iscove’s modified Dulbecco’s medium (IMDM), Dulbecco’s modified Eagle’s medium (DMEM), fetal bovine serum (FBS), 0.05% trypsin, 0.25% trypsin, glutamine, penicillin, and streptomycin were purchased from Gibco BRL (Grand Island, NY, USA). Lipopolysaccharide (LPS E. coli 026:B6), rapamycin (R0395), 3-[4,5-dimethylthiazol-2-yl]-2,5-diphenyltetrazolium bromide (MTT), and chloroquine (CQ, C6628) were obtained from Sigma Chemical Co. (St. Louis, MO, USA). 3-Methyladenine (3-MA) was purchased from Selleck. TNF-α, IL-1β, IL-6, and IL-10 ELISA kits were from eBioscience. TRIzol reagent was from Invitrogen Co. (Carlsbad, CA, USA). PrimeScript™ RT reagent Kit with gDNA Eraser and SYBR Premix EX Taq™ II were purchased from Takara Biotechnology (Dalian, China).

Antibodies used in this study include anti-PI3KI (1:1000; CST, 4292 s), anti-AKT (1:1000; CST, 9272 s), anti-p-AKT (Ser473) (1:1000; CST, 9271 s), anti-MTOR (1:1000; CST, 2972 s), anti-p-MTOR (Ser2448) (1:1000; CST, 2971 s), anti-p70S6K (1:1000; CST, 5707 s), anti-p-p70S6K (Thr389) (1:1000; CST, 9234 s); anti-Beclin-1 (1:1000; CST, 3495 s), anti- SQSTM1 (1:1000; CST, 5114), anti-Vps34 (1:500; Invitrogen, 382100), LC3 (1:1000; MBL, PM036), LAMP2 (1:2000; Sigma, L0668), anti-CSTE (1:1000; Abcam, ab36996), anti-ATG5 (1:1000; MBL, PM050), β-actin (1:1000; Santa Cruz, sc-47778), β-tubulin (1:1000; Sigma, T4026), LC3 (1:200, MBL, M152-3, immunocytochemistry), and Iba-1(1:800; Millipore, MABN92).

### Cell culture

Primary microglia were prepared from hippocampus and cerebral cortex of postnatal day 1 Sprague-Dawley (SD) rats. Briefly, both the hippocampus and cerebral cortex were dissociated by treatment with 0.25% trypsin. The cells were maintained in DMEM with 10% FBS at 37 °C in a 5% CO_2_ humidified atmosphere. After 14 days in culture, the flasks were shaken to separate microglia from mixed glial cultures. The purity of primary microglia was greater than 95%.

The murine microglial cell line N9 was a kind gift from Dr. J.M. Wang (Laboratory of Molecular Immunoregulation, Center for Cancer Research, National Institutes of Health, USA). N9 microglial cells were cultured in IMDM supplemented with 5% heat-inactivated FBS, 2 mM glutamine, 100 μg/ml streptomycin, and 100 U/ml penicillin at 37 °C in humidified 5% CO_2_. Cells were subcultured with a 1:4 split ratio and used within 8 passages.

### Animals

Male C57BL/6 mice weighing 18–22 g were obtained from Weitonglihua Company (Beijing, China). All animals were kept under standard housing conditions on a 12 h/12 h modified light/dark cycle with free access to water and food. All the experiments were carried out according to the Regulations for the Administration of Affairs Concerning Experimental Animals of China.

### Drug administration

Intracerebroventricular injections (i.c.v.) were made under anesthesia with 1% pentobarbital sodium by intraperitoneal injection (i.p.) at a dose of 80 mg/kg. Stereotaxic bregma coordinates were − 0.46 mm anteroposterior, 1.0 mm lateral (left side), and − 2.5 mm dorsoventral [32]. Injections were carried out using a Hamilton syringe. The MTOR inhibitor rapamycin was first dissolved in DMSO to prepare a 10-mM stock solution. Thereafter, it was diluted with ACSF (artificial cerebrospinal fluid) to reach concentrations of 0.125, 0.25, or 0.5 mM. To produce mouse models of neuroinflammation, 5 μg LPS [33] was diluted in ACSF at a concentration of 2.5 μg/μL and administered by intracerebroventricular injection. Sham operation mice received the vehicle containing an equal volume of diluent and DMSO lacking rapamycin and LPS.

The mice were administered with different doses of rapamycin at 0.25, 0.5, and 1 nmol [34]. The needle was kept in place for another 3 min to allow diffusion. After 15 min, LPS (5 μg) was injected at a speed of 1 μL/min and the injection needle was left for another 3 min [35, 36]. The mice were sacrificed 24 h after i.c.v. injection. Samples were stored at − 80 °C until ELISA and immunohistochemistry as described below.

### Cell viability assay

Cell viability was measured by the MTT reduction assay. In brief, N9 microglial cells or SH-SY5Y neuroblastoma cells were seeded in 96-well plates at a density of 1 × 10^4^ cells per well for 24 h and treated with various reagents for the indicated time periods. After various treatments, the media were removed and the cells were incubated with MTT (0.25 mg/mL) for 3 h at 37 °C. The formazan crystals in the cells were solubilized with DMSO. Finally, the absorbance of MTT formazan was obtained using a plate reader at 490 nm (Synergy HT, BioTek, USA).

### Nitrite assay

Accumulation of nitrite (NO_2_^−^), an indicator of NO production, in culture supernatant fluids was evaluated by the Griess reaction [12]. N9 microglial cells were seeded in 96-well plates at 2 × 10^4^ cells per well and treated with different concentrations of LPS for 24 h. Fifty microliters of culture supernatant was mixed with 50 μL Griess reagent at room temperature for 15 min. The absorbance was obtained at 540 nm using a plate reader (Synergy HT, BioTek, USA).

### Preparation and application of conditioned medium from microglia

N9 microglial cells were pretreated with rapamycin (100 nM) for 2 h and then stimulated with LPS (1 μg/mL) for 24 h. The culture media were collected and clarified by centrifugation at 12,000*g* for 5 min as conditioned media (CM). The conditioned media were then added to SH-SY5Y cells, which were further incubated at 37 °C for 24 h. Cell viability was measured by MTT assay.

### Preparation and transfection of plasmids

Plasmids were constructed as follows. cDNAs encoding LC3 and DFCP1 were cloned into pEGFP-C2 vector between XhoI and SacII sites. cDNA encoding WIPI2 was cloned into RFP-N1 vector between Hind III and Bam HI sites. cDNA encoding Vps34 was cloned into pCDH-EF1-mCherry-T2A-Puro vector between EcoRI and BamHI sites. N9 microglial cells were cultured in a 24-well plate containing coverslips at a density of 5 × 10^4^ cells per well. At 70% confluence, the culture medium was replaced with 350 μL complete medium without penicillin-streptomycin. Cells were transfected with plasmids using Lipofectamine 2000 (Life Technologies, 11668027) for 24 h according to the manufacturer’s instructions. Thereafter, cells were treated with LPS (1 μg/mL) for the indicated time.

### Lentivirus packaging and establishment of stable N9 microglial cell lines

Lentiviruses were produced in 293FT cells by co-transfecting pLnV, pVSVG, and pCDH-EF1-Vps34-mCherry-T2A-Puro-SV40 Poly A using Lipofectamine 2000 transfection reagent. The supernatant was harvested at 48 h and 72 h after transfection. Filtered viral supernatants were used to infect N9 microglial cells and maintained in medium containing 3 μg/mL puromycin 48 h after infection. Control cells were infected with virus produced from the pCDH-EF1-mCherry-T2A-Puro empty vector. The transcription and expression of Vps34 in N9 cells were detected by western blotting.

### Western blotting

All cells and tissues were homogenized and lysed in RIPA buffer (Sigma-Aldrich) containing 1 mM phenylmethyl-sulfonyl fluoride (Abcam) and Complete Protease Inhibitor Cocktail (Roche) on ice for 30 min. Lysates were centrifuged at 12,000 rpm for 15 min at 4 °C, and total protein concentrations of the supernatant fractions were detected by a BCA Protein Assay Kit (Thermo Fisher Scientific). Equal amounts of protein were separated by 8–12% SDS-PAGE gels and then transferred onto 0.22-μm pure Nitrocellulose blotting membrane (Pall Life Sciences). The membranes were blocked with 5% skimmed milk, and then incubated overnight with primary antibodies at 4 °C. The membranes were washed with TBST 4 times (5 min/time) then incubated with HRP-conjugated secondary antibodies. The immunoreactive bands were visualized by enhanced chemiluminescence (ECL).

### Detection of lysosomal pH in N9 microglial cells

The pH of lysosomes in N9 microglial cells was detected using LysoSensor Green DND-189 (Invitrogen, L7535). N9 Microglial cells were seeded in 96-well plates at a density of 1 × 10^4^ cells per well for 24 h. After treatment with LPS for 6–24 h or 100 nM bafilomycin A1 (Baf A1) for 6 h, the media were removed from the wells and replaced with medium containing LysoSensor Green (1 μM). The cells were further incubated for 1 h under growth conditions and then washed with PBS. Finally, the fluorescence was read at an excitation wavelength of 443 nm and an emission wavelength of 505 nm (Thermo, VARIOSKAN FLASH).

### Immunocytochemistry

N9 microglial cells were seeded in an 8-well plate (Lab-Tek) at a density of 2.5 × 10^4^ cells per well for immunostaining. After treatment with LPS, cells were fixed in 4% paraformaldehyde for 10 min at room temperature, followed by permeabilization with 100 μg/mL cold digitonin (Sigma, D141) for 10 min at 4 °C. After blocking with 5% goat serum for 1 h at room temperature, the primary antibody was applied overnight at 4 °C. After washing with PBST 3 times, the cells were incubated with Alexa Fluor® 488–conjugated goat anti-mouse secondary antibody for 1 h at room temperature in a dark place and washed. Cells were stained with Hoechst 33342 for 10 min followed by extensive washes with PBST. Images were taken on a confocal microscope (LSM 710, Zeiss) then analyzed by image J. Pearson’s correlation coefficients were calculated using the Coloc2 plugin to analyze the colocalization of DCFP1 and WIPI2 [37].

### Brain tissue preparation and immunohistochemistry

Mice were anesthetized with 1% pentobarbital sodium by intraperitoneal injection (i.p.) at a dose of 80 mg/kg (*n* = 4/group) and then transcardially perfused with 0.9% saline followed by 4% paraformaldehyde (PFA). Their brains were postfixed in 4% PFA for 24 h and immersed in 20% and 30% sucrose at 4 °C. Each brain was embedded in OTC medium and sectioned on a freezing microtome (Leica CM1950, Germany) at a thickness of 20 μm in each region. After cutting, sections were stored at − 20 °C until required for further processing.

Sections were removed from storage at − 20 °C and washed 4 times in 0.01 M PBS for 5 min. Antigen retrieval was performed by microwave heating of the sections in 10 mM trisodium citrate buffer (pH 6.0, 85 °C) for 30 min. The sections were washed twice in PBS and treated with 3% hydrogen peroxide in H_2_O for 5 min to inhibit endogenous peroxidase activity. Then, the sections were blocked with 2% goat serum for 1 h in a humid chamber at 37 °C and incubated with monoclonal mouse anti-Iba1 primary antibody (Millipore, Billerica, USA) overnight at 4 °C. The sections were labeled with a secondary antibody (biotinylated goat anti-mouse) and incubated with streptavidin-coupled horseradish peroxidase for 1 h at 37 °C, and visualized by incubating with DAB (ZSGB-BIO, China) for 10–60 s. Lastly, the sections were dehydrated using graded ethanol, cleared in 100% xylene and covers-lipped with Permount TM Mounting Medium [38]. Images were analyzed and quantified using Image J software to measure the number of Iba1 positive cells and to trace the microglial processes for length measurement. Two coronal sections from at least three animals were used for quantification [39, 40].

### Quantitative real-time reverse transcription polymerase chain reaction (qRT-PCR)

Total RNA was extracted from N9 microglial cells using TRIzol reagent (Invitrogen, CA), and reverse transcription was performed with a PrimeScript™ RT reagent Kit. Quantitative real-time PCR was performed using a SYBR Premix EX Taq™ II Kit. The comparative Ct (2^−ΔΔCt^) method was used to analyze the relative expression of mRNAs normalized to *Gapdh*. The qRT-PCR primers are listed in Table 1. The qRT-PCR conditions were initial denaturation, 95 °C for 30s; annealing, 64 °C for 60 s; extension, 72 °C for 60 s; 35 cycles.

**Table 1:** Primer sequences for qRT-PCR

Gene	Forward primer	Reverse primer
*Tnf-α*	5′ AGACCCTCACACTCAGATCATCTTC 3′	5′ TTGCTACGACGTGGGCTACA 3′
*Il-6*	5′ TAGTCCTTCCTACCCCAATTTCC 3′	5′ TTGGTCCTTAGCCACTCCTTC 3′
*Inos*	5′ GGCAAACCCAAGGTCTACGTT 3′	5′ GAGCACGCTGAGTACCTCATTG 3′
*Gapdh*	5′ GGCAAATTCAACGGCACA 3′	5′ GAGCACGCTGAGTACCTCATTG 3′

### Enzyme-linked immunosorbent assay

Hippocampi from each group were homogenized with 10 volumes of ice-cold saline and centrifuged at 12,000*g* for 10 min. Supernatants were transferred to a new tube and stored at − 80 °C until measured. The samples were added into each well of pre-coated plates, and all steps were performed according to the manufacturer’s guidelines.

### Transmission electron microscopy

Following experimental treatments, N9 microglial cells were fixed with 2.5% glutaraldehyde buffer for 2 h at 4 °C. Subsequently, samples were washed 3 times in PBS and then postfixed in 1% osmium tetroxide for 2 h, processed through a graded ethanol series, embedded in araldite and polymerized at 37, 45, and 60 °C for 24 h at each temperature. Ultrathin sections (70 nm) were cut with a diamond knife on a Reichert Ultracut S and recovered on Cu grids and then were stained with uranyl acetate and lead citrate. Finally, the sections were observed under a transmission electron microscope (H-7650B, Hitachi, Japan) [41].

### Statistical analysis

All values are expressed as mean ± SEM. Statistical analysis was carried out using SPSS 17.0 software for Windows (SPSS Inc., USA). Data were analyzed with one-way ANOVA followed by post hoc Tukey’s HSD test to compare the differences among three or more groups. Student’s *t* test was used to compare the difference between two experimental groups. *P* < 0.05 was considered as significant.

## Results

### LPS inhibits autophagy in microglia

Firstly, to explore the role of autophagy in the microglial neuroinflammatory response, N9 microglial cells were treated by LPS for 24 h. The data showed that LPS significantly increased the levels of nitrite (a stable oxidized product of NO) in a dose-dependent manner (0.001–10 μg/mL) without influencing the cell viability (Additional file 1: Figure S1a-b), which suggests that LPS triggered an inflammatory reaction in N9 microglial cells. Western blot analysis showed that 1 μg/mL LPS markedly increased the expression of iNOS in N9 microglial cells from 6 to 24 h (Additional file 1: Figure S1c-d). Thus, 1 μg/mL of LPS was chosen for the following studies.

**Fig. 1:**
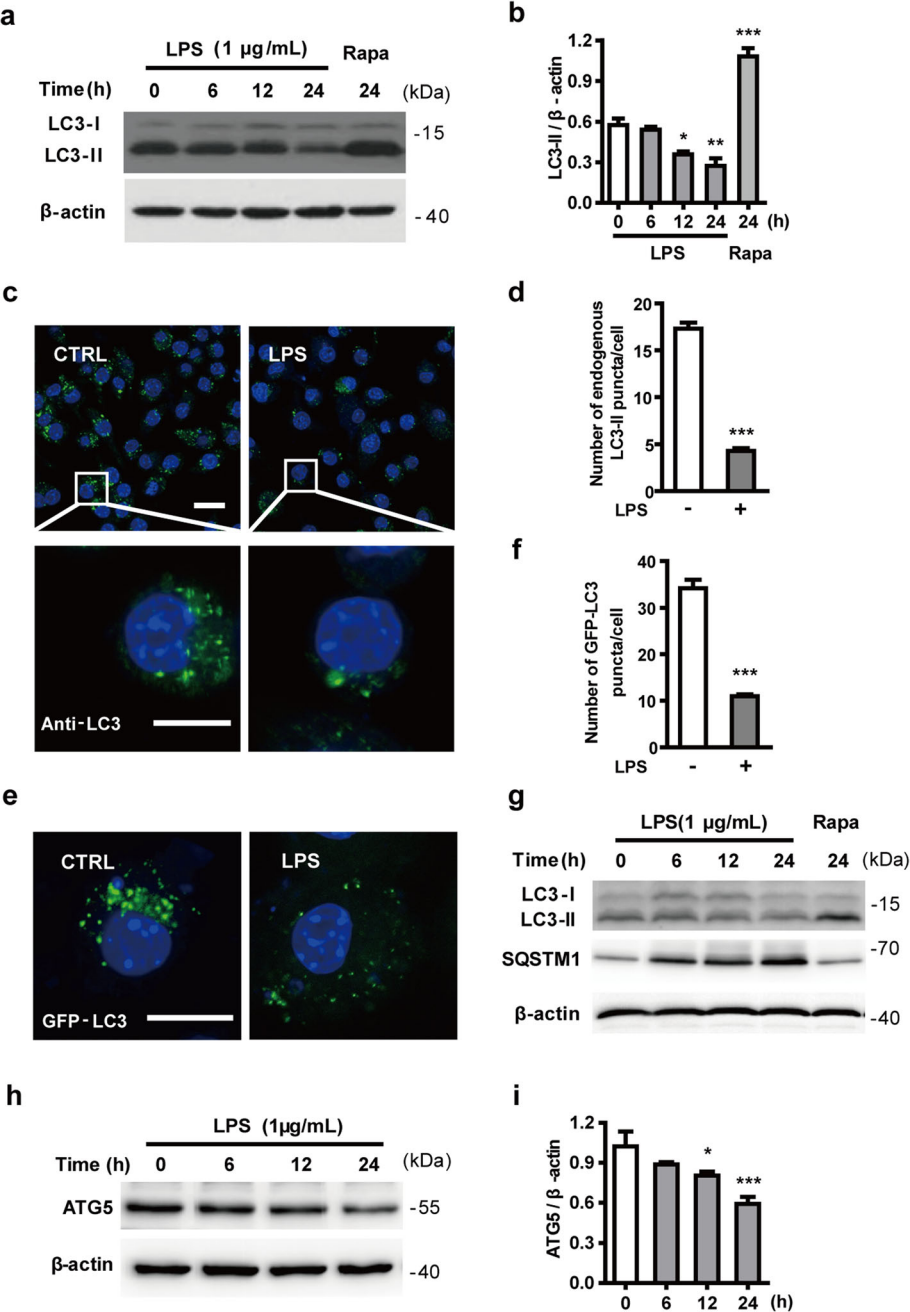
LPS inhibits autophagy in microglia. **a** The level of LC3-II, an autophagosome marker, declines in LPS-activated microglial cells. N9 microglial cells were stimulated with 1 μg/mL LPS for 6, 12, and 24 h. Cells were lysed and the levels of LC3-II (lipidated LC3) were analyzed by western blotting and quantified (**b**). **c** N9 microglial cells were treated with or without 1 μg/mL LPS for 24 h. PFA-fixed cells were stained with an antibody against LC3 and assessed by confocal microscopy to detect endogenous LC3-positive puncta (autophagosomes). Immunofluorescence images show the LC3-positive puncta (green) in CTRL- and LPS-treated cells. Nuclei are stained with DAPI (blue). Scale bar: upper: 20 μm, lower: 10 μm. **d** Statistical analysis of the number of endogenous LC3-positive puncta per cell in (**c**), from 3 independent experiments with at least 50 cells per treatment. **e** N9 microglial cells were transfected with GFP-LC3 plasmid for 24 h and treated with or without 1 μg/mL LPS for 24 h. GFP-LC3 puncta, representing autophagosomes, were observed by confocal microscopy. Representative images show GFP-LC3 (green) and DAPI (blue) in the CTRL and LPS-treated cells. Scale bar: 10 μm. **f** Statistical analysis of the number of GFP-LC3 positive puncta per cell in (**e**). **g** Primary microglial cells, isolated from rat brains were treated with LPS (1 μg/mL) for 6, 12, and 24 h or rapamycin (100 nM) for 24 h. Cells were lysed and the levels of LC3-II (lipidated LC3) and SQSTM1 were analyzed by western blotting. **h** N9 microglial cells were exposed to 1 μg/mL LPS for 6 h, 12 h, and 24 h. The expression of ATG5 was detected by western blotting and quantified (**i**). Data are presented as mean ± SEM. ^*^p < 0.05, ^**^p < 0.01, ^***^p < 0.001 vs control

Next, to clarify the effect of LPS-induced microglial neuroinflammation on autophagy, we examined the expression of LC3-II, which is required on the developing autophagosome membrane [42]. As shown in Fig. 1a, b, the LC3-II levels declined in N9 microglial cells after exposure to LPS for 12 h and 24 h compared with the control group. The expression of LC3-II was significantly increased by treatment with 100 nM rapamycin for 24 h, which indicates that rapamycin can enhance the autophagy activity of N9 microglial cells. The effect of LPS on the formation of endogenous LC3-positive autophagosomes was assessed by staining with LC3 antibody. As shown in Fig. 1c, d, the number of endogenous LC3-positive puncta per cell was significantly lower in LPS-stimulated N9 microglial cells compared with cells without LPS. N9 microglial cells were also transiently transfected with a GFP-LC3 plasmid. At 24 h after transfection, cells were treated with or without LPS for another 24 h before analysis by immunocytochemistry. The confocal images show that fewer GFP-LC3-positive puncta (autophagosomes) were formed in LPS-treated N9 microglial cells than control cells (Fig. 1e, f). In primary microglial cells, LPS reduced the expression of LC3-II (similar to N9 microglial cells), and increased the expression of SQSTM1 (Fig. 1g). In addition, the expression of the autophagy-related protein ATG5 decreased after LPS treatment in N9 microglial cells (Fig. 1h, i). These results indicate that autophagy is impaired in LPS-stimulated microglial cells.

### LPS suppresses autophagic flux by inhibiting autophagosome formation in N9 microglial cells

**Fig. 2:**
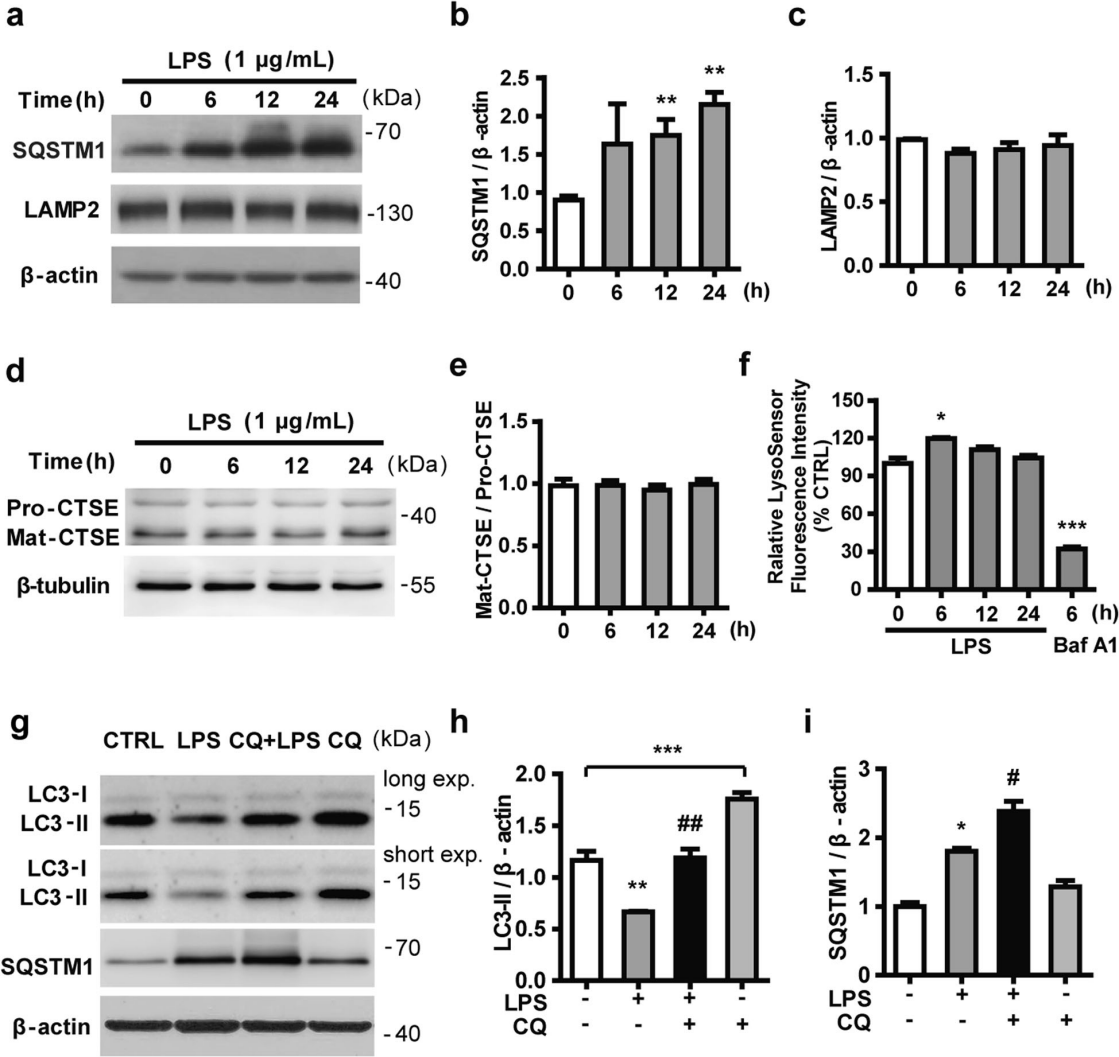
LPS suppresses the formation of autophagosomes without influencing the function of lysosomes. **a** N9 microglial cells were exposed to 1 μg/mL LPS for 6 h, 12 h, and 24 h. The expressions of SQSTM1, an autophagy substrate, and LAMP2, a lysosome membrane protein, were detected by western blotting and quantified (**b**, **c**). **d** Levels of Cathepsin E (CTSE) were determined by western blotting in microglial cells treated with LPS for 6 h, 12 h, and 24 h, and quantified (**e**). **f** N9 microglial cells were treated with 1 μg/mL LPS for 6 h, 12 h, and 24 h, or with 100 nM bafilomycin A1 for 6 h. The fluorescence intensity of LysoSensor was recorded with a 443-nm excitation filter and a 505-nm emission filter. **g** N9 microglial cells were treated with LPS in the presence or absence of 10 μΜ chloroquine (CQ) for 24 h. The expression of autophagy-related proteins was measured by western blotting and quantified (**h, i**). Data are presented as mean ± SEM. ^*^p < 0.05, ^**^p < 0.01, ^***^p < 0.001 vs control; ^#^*p* < 0.05, ^##^*p* < 0.01 vs CQ

The autophagy adaptor protein SQSTM1/p62 is localized to autophagosomes via interaction with LC3. It is also a substrate for selective autophagy and is constantly degraded by the autophagy-lysosome system. Accordingly, the elimination of cellular autophagy will result in an abnormal accumulation of SQSTM1 [43]. As shown in Fig. 2a, b, SQSTM1 gradually accumulated in N9 microglial cells exposed to LPS. To identify whether SQSTM1 accumulation is due to lysosomal dysfunction, the expression of LAMP2 protein, a lysosomal membrane marker, was examined by western blotting. The results showed that the expression of LAMP2 was not significantly changed compared with the control group after treatment with LPS for 6 h to 24 h (Fig. 2a, c). To assess whether LPS impaired the lysosomal activity, we examined the expression of Cathepsin E, a lysosome-related enzyme, and lysosomal acidification. Western blotting analysis showed that LPS had no effect on the expression of Cathepsin E (Fig. 2d, e). Lysosomal acidification was probed with LysoSensor Green, which undergoes a pH-dependent increase in fluorescence intensity upon acidification. Treatment with LPS for 6 h induced a transient increase of fluorescence intensity, which returned to the baseline from 12 h to 24 h, suggesting that LPS did not attenuate lysosomal acidity in N9 microglial cells (Fig. 2f). We reasoned that the accumulation of SQSTM1 might not be caused by lysosomal dysfunction. In order to elucidate whether the LPS-induced accumulation of SQSTM1 and decreased LC3 expression may result from a defect in the upstream steps of autophagy, we treated N9 microglial cells with chloroquine (CQ, 10 μM), an agent that impairs lysosomal acidification. As shown in Fig. 2g–i, CQ was effective in preventing the degradation of LC3-II, as judged from the significant accumulation of LC3-II. In the presence of CQ, LPS reduced the LC3-II level, but caused a further accumulation of SQSTM1. Collectively, these data demonstrated that in LPS-activated microglia, the decrease in LC3 was due to impaired autophagosome formation rather than lysosomal dysfunction.

### LPS inhibits autophagosome formation via Vps34

**Fig. 3:**
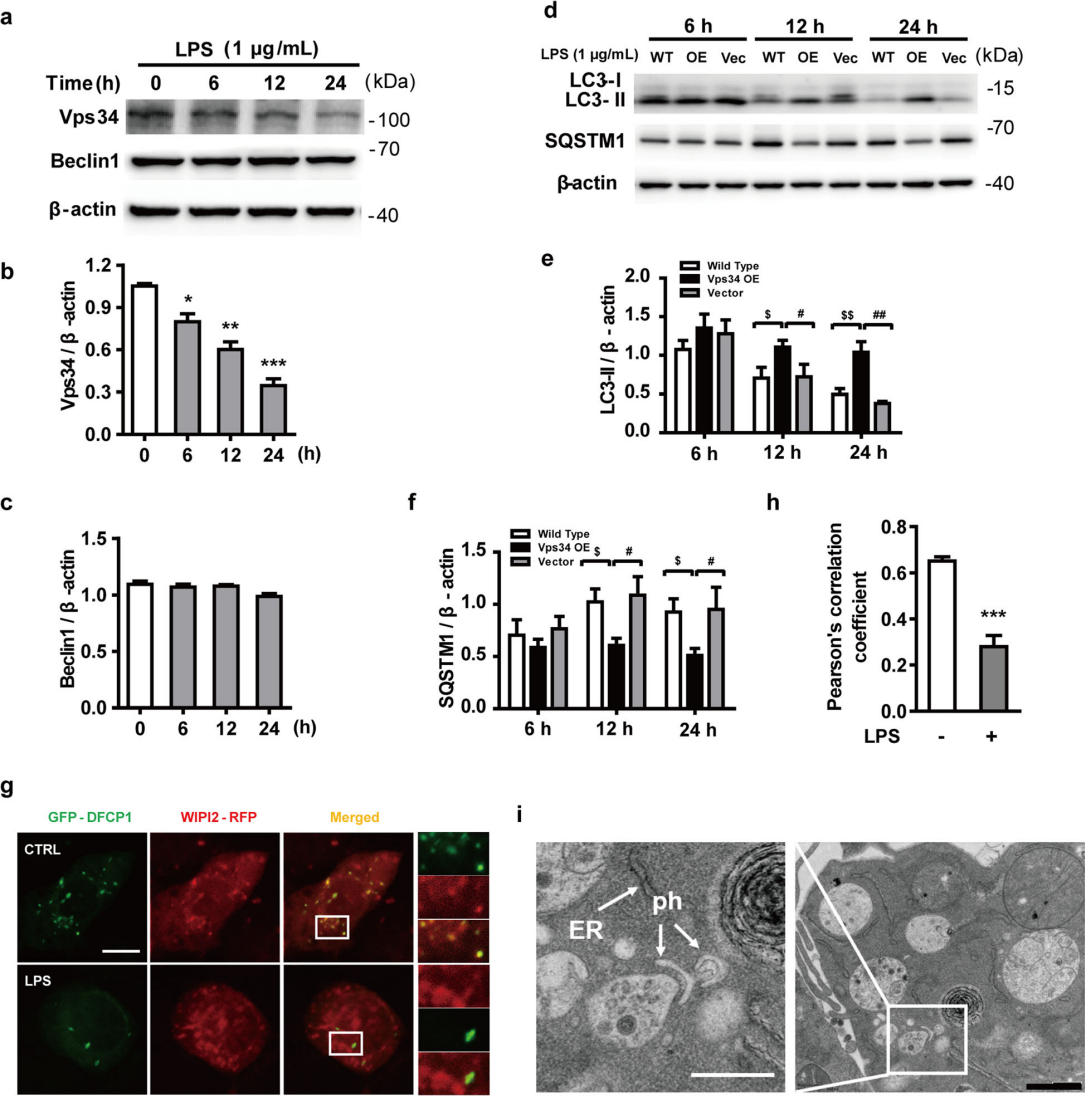
LPS reduces the expression of Vps34, resulting in the inhibition of omegasome formation and phagophore formation in N9 microglial cells. **a** The protein levels of Vps34 and Beclin1 were detected by western blotting in LPS-treated N9 microglial cells and quantified (**b**, **c**). **d** The expression of LC3 and SQSTM1 were detected by western blotting in Vps34 overexpressing N9 microglial cells after treatment with 1 μg/mL LPS for 6 h, 12 h, and 24 h, and quantified (**e**, **f**). **g** N9 microglial cells were transfected with GFP-DFCP1 and RFP-WIPI2 plasmids for 24 h and then treated with or without 1 μg/mL LPS for 12 h, and visualized by confocal microscopy. Immunofluorescence images show the colocalization of DFCP1 (green) with WIPI2 (red) in the CTRL and LPS-treated cells. The boxed areas are enlarged in the right panels. Scale bar: 10 μm. **h** Pearson’s correlation coefficient for colocalization of DFCP1 with WIPI2 was calculated by Image J. Data are presented as mean ± SEM of three independent experiments. **i** Representative TEM images of N9 microglial cells cotreated with 100 nM rapamycin and 1 μg/mL LPS for 12 h. Arrows indicate phagophores (ph) and endoplasmic reticulum (ER). Scale bar: 500 nm (white), 1 μm (black). Data are presented as mean ± SEM. ^*^p < 0.05, ^**^p < 0.01, ^***^p < 0.001 vs control; ^$^*p* < 0.05, ^$$^*p* < 0.01 vs wild type; ^#^*p* < 0.05, ^##^ *p* < 0.01 vs vector

The upstream steps of autophagy involve the formation and maturation of omegasomes, mediated by the class III phosphatidylinositol 3-kinase (PtdIns3K) complex [44]. We examined the expression of two subunits of this complex, Beclin1 and Vps34, in LPS-stimulated microglia. LPS did not influence the expression of Beclin 1 but significantly decreased the levels of Vps34 (Fig. 3a–c). These observations led us to postulate that increased Vps34 expression may regulate autophagic impairment and contribute to the improvement of neuroinflammation. To directly ascertain the role of Vps34 in LPS-induced N9 microglial cells, we generated an N9 microglial cell line stable overexpressing Vps34 by lentivirus infection (Additional file 2: Figure S2a). After treatment with 1 μg/mL LPS for 6 to 24 h, cells overexpressing Vps34 showed a robust expression of LC3-II compared with those transduced with mCherry vector or wild-type N9 microglial cells (Fig. 3d, e). The overexpression of Vps34 not only successfully attenuated the accumulation of the autophagy substrate SQSTM1 (Fig. 3d, f), but also reduced the mRNA levels of inflammatory cytokines (Additional file 2: Figure S2b). These results indicated that LPS-induced autophagic impairment might be related to Vps34 downregulation. Previous studies reported that Vps34 generates phosphatidylinositol 3-phosphate (PtdIns3P, PI(3)P) on the ER to regulate various cellular processes by recruiting specific protein effectors to target membranes [16]. To clarify the role of PI(3)P in LPS-stimulated N9 microglia, the cells were transiently transfected with GFP-DFCP1 and WIPI2-RFP plasmids. In mammalian cells, PI(3)P-enriched ER subdomains, known as omegasomes, act as platforms for autophagosome formation upon autophagy induction [16]. WIPI2, a WD40 repeat–containing PI(3)P–binding protein, functions at the step at which omegasomes progress to autophagosomes [11]. Twenty-four hours after transfection, N9 microglial cells were treated with or without LPS for 12 h and visualized by confocal microscopy. The fluorescence images and colocalization analysis based on Pearson’s correlation coefficient showed that GFP-DFCP1 colocalized extensively with WIPI2-RFP in the control group. However, the colocalization of GFP-DFCP1 and WIPI2-RFP was greatly diminished in LPS-induced N9 microglial cells (Fig. 3g, h), which indicates that the reduction of PI(3)P impeded the process of omegasome expansion into phagophores. Since omegasome maturation is inhibited in LPS-treated cells, it is hard to catch phagophores (Additional file 3: Figure S3a). Therefore, we used rapamycin to induce autophagy in N9 microglial cells. Phagophores were found close to the ER by transmission electron microscopy (TEM) after cotreatment with LPS and rapamycin (Fig. 3i), but no double-membraned autophagosome structures were observed in LPS-treated cells (Additional file 3: Figure S3b). These results indicate that LPS reduces autophagosome formation at a very early stage of biogenesis. Together, these data suggest that LPS inhibits the formation of omegasomes, thereby leading to impaired autophagy.

### LPS activates the PI3KI/AKT/MTOR signaling pathway in N9 microglial cells

**Fig. 4:**
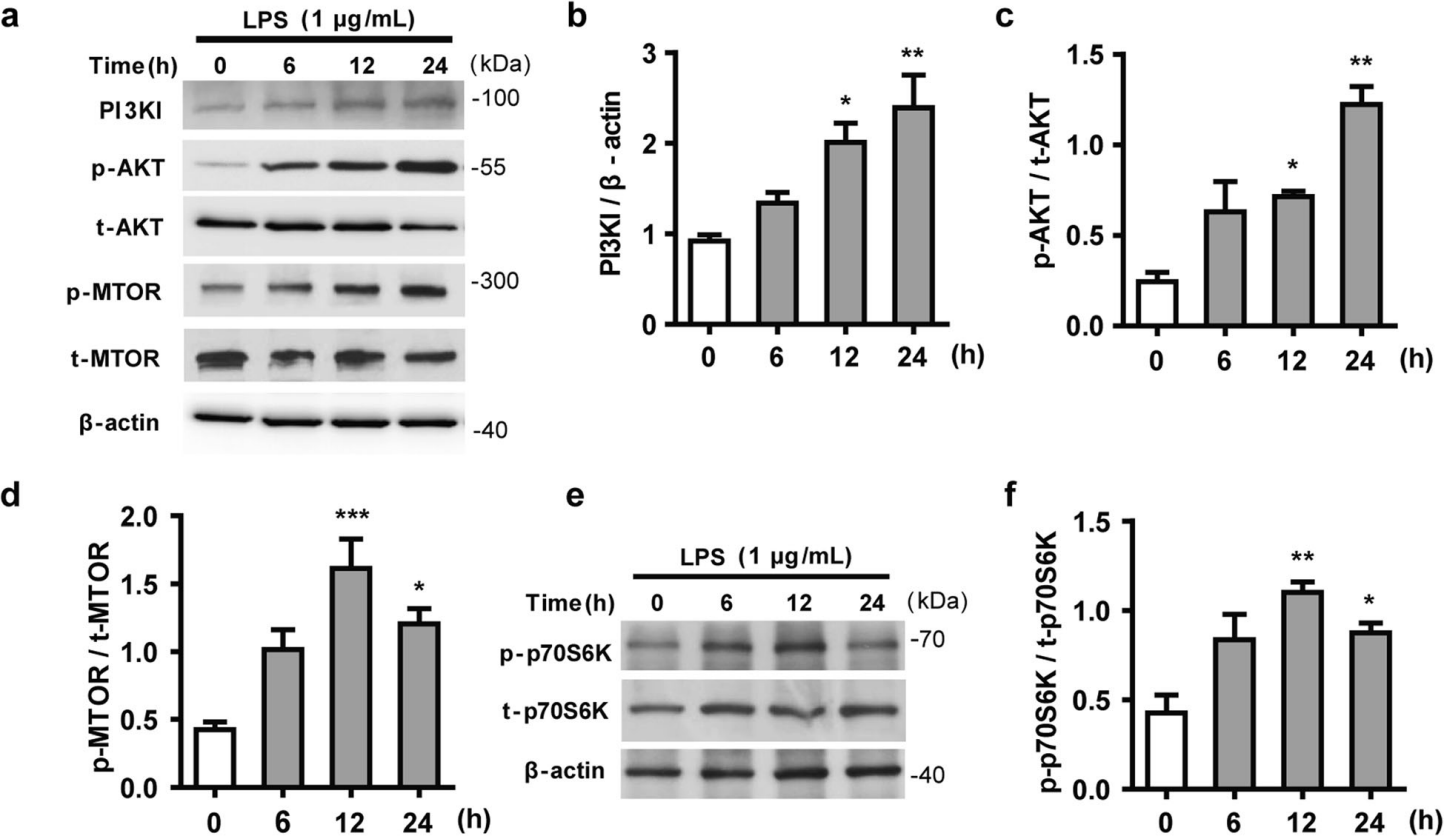
LPS inhibits autophagosome formation in N9 microglial cells by activating the PI3KI/AKT/MTOR pathway. Microglial cells were treated with 1 μg/mL LPS for the indicated times. The protein levels of PI3KI, p-AKT, AKT, p-MTOR, MTOR (**a**), p-p70S6K and p70S6K (**e**) were analyzed by western blotting and quantified (**b**–**d**, **f**). Data are presented as mean ± SEM. ^*^p < 0.05, ^**^p < 0.01, ^***^p < 0.001 vs control

MTOR signaling is recognized as a major negative regulator of autophagy at its early stage. To explore the deficiency in autophagosome formation caused by LPS in N9 microglial cells, we analyzed upstream MTOR signaling. As shown in Fig. 4a–c, LPS induced a noticeable increase in the levels of PI3KI and phosphorylated AKT from 6 to 24 h. The activation of AKT via phosphorylation leads to the activation of MTOR, which results in the inhibition of autophagy [45]. As shown in Fig. 4a, d–f, MTOR activation was significantly increased after LPS treatment. Western blotting revealed a higher level of phosphorylated MTOR in parallel with increased phosphorylation of one of its substrates, p70S6K. These results confirmed that the activation of the PI3KI/AKT/MTOR signaling pathway was associated with suppression of autophagy induction and autophagosome formation in LPS-activated microglia.

### LPS-induced the production of pro-inflammatory cytokines is reversed by activation of autophagy both in vitro and in vivo

**Fig. 5:**
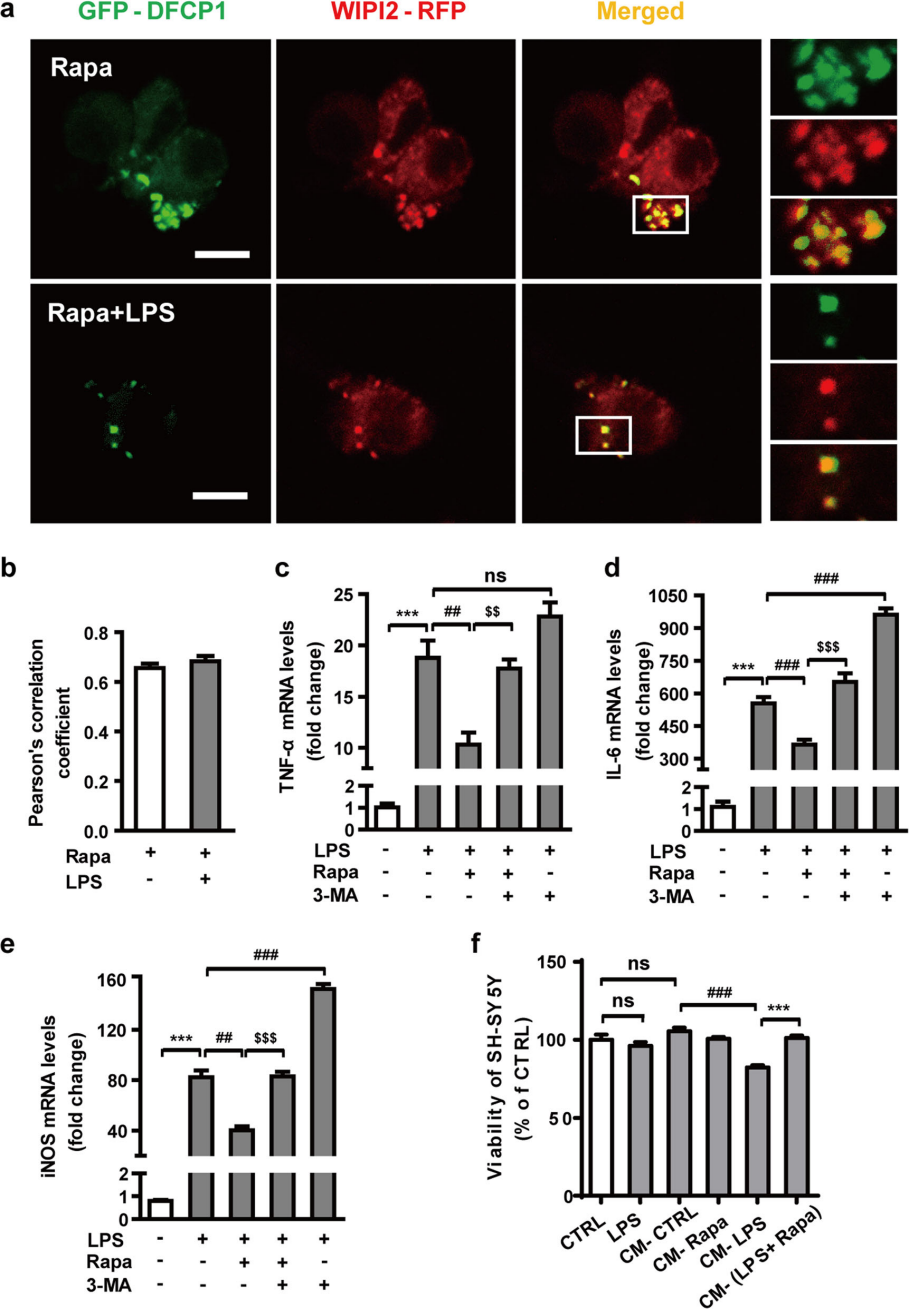
Rapamycin alleviates the LPS-induced neuroinflammation in N9 microglial cells. **a** Treatment of N9 microglial cells with rapamycin enhances autophagy and facilitates the colocalization of GFP-DFCP and WIPI2-RFP even after stimulation with 1 μg/mL LPS for 12 h. Microglial cells were transfected with GFP-DFCP1 and RFP-WIPI2 plasmids for 24 h and then treated with 100 nM rapamycin with or without 1 μg/mL LPS for 12 h. Images were obtained by confocal microscopy. Scale bar: 10 μm. **b** Pearson’s correlation coefficient for colocalization of DFCP1 with WIPI2 was calculated by Image J. N9 microglial cells were treated with vehicle, LPS (1 μg/mL), rapamycin (100 nM) + LPS, rapamycin + LPS + 3-MA (2.5 mM), or LPS + 3-MA for 6 h. The levels of mRNAs encoding the proinflammatory cytokines TNF-α (**c**), IL-6 (**d**), and iNOS (**e**) were analyzed by qRT-PCR. Data are presented as mean ± SEM. ^***^p < 0.001 vs control; ^##^*p* < 0.01, ^###^*p* < 0.001 vs LPS; ^$$^*p* < 0.01, ^$$$^*p* < 0.001 vs cotreatment with LPS and rapamycin. **f** Effects of different conditioned media on the viability of SH-SY5Y cells assessed by MTT. Conditioned media (CM) were obtained from N9 microglial cells treated with or without LPS and rapamycin as indicated. Data are presented as mean ± SEM. ^###^*p* < 0.001 vs CM-CTRL; ^***^p < 0.001 vs CM-LPS

Next, we sought to test the hypothesis that the LPS-induced neuroinflammation was associated with MTOR-mediated autophagy inhibition. We used rapamycin, an MTOR inhibitor and inducer of autophagy, to increase autophagy activity in N9 microglial cells. N9 microglial cells were pretreated with rapamycin for 2 h and then exposed to LPS and rapamycin for 6 h. As shown in Fig. 5a, b, rapamycin treatment facilitated the colocalization of GFP-DFCP1 and WIPI2-RFP even after 12 h of LPS stimulation. Furthermore, the transcript levels of various inflammatory cytokines were measured by qRT-PCR using mRNA extracted from microglial cells. Compared with LPS alone, rapamycin significantly downregulated the levels of transcripts encoding the pro-inflammatory cytokines TNF-α, IL-6, and iNOS (Fig. 5c–e). These results indicate that rapamycin-induced activation of autophagy significantly reduces LPS-induced microglial activation. We also blocked autophagy by treating N9 microglial cells with the Vps34 inhibitor 3-methyladenine (3-MA). 3-MA not only promoted the LPS-induced release of inflammatory cytokines, but also reversed the anti-inflammatory effect of rapamycin. In addition, we treated SH-SY5Y neuroblastoma cells with conditioned medium from N9 microglial cells to investigate whether autophagy is able to inhibit microglia-mediated neuroinflammation. N9 microglial cells were pretreated with rapamycin for 2 h and then incubated with LPS and rapamycin for 24 h. The culture media were collected as conditioned medium and then transferred to SH-SY5Y neuroblastoma cells and incubated for 24 h. Compared with the control conditioned medium (CM-CTRL), the conditioned medium from LPS-stimulated microglia (CM-LPS) caused an approximately 20% reduction in the viability of the SH-SY5Y cells (Fig. 5f). However, this effect was reversed by rapamycin pretreatment without influencing the basal level of cell viability. In addition, the results showed that LPS was not directly cytotoxic to SH-SY5Y. These results suggest that rapamycin-induced autophagy has a neuroprotective effect, which is mediated predominantly by suppressing the neurotoxicity associated with excessive microglial pro-inflammatory response.

**Fig. 6:**
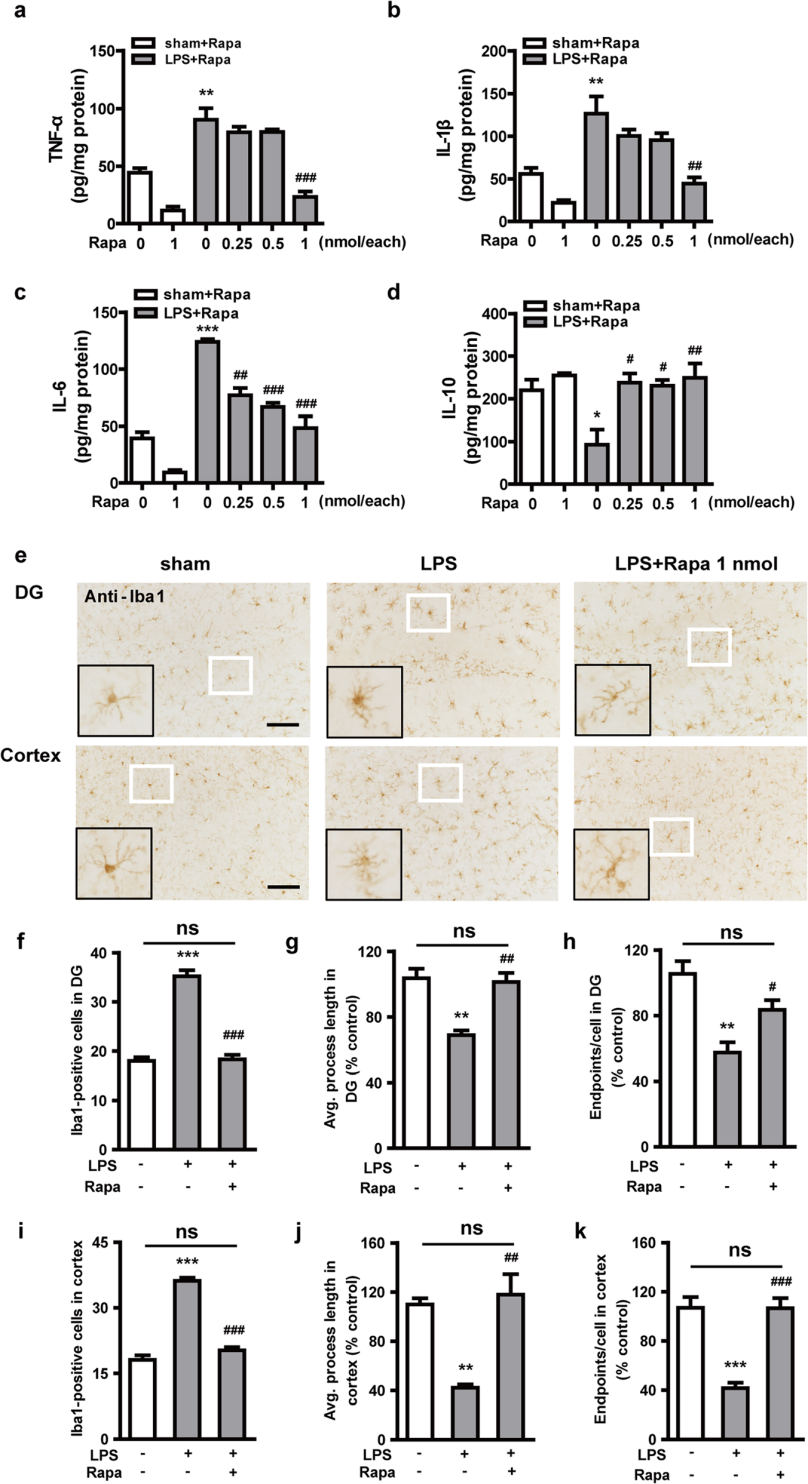
Rapamycin alleviates LPS-induced neuroinflammation in hippocampus and cortex of mice. Different doses of rapamycin (0.25, 0.5, 1 nmol for each mouse) were administered via intracerebroventricular injection 15 min before 5 μg LPS administration. The hippocampus levels of the pro-inflammatory cytokines TNF-α (**a**), IL-1β (**b**), and IL-6 (**c**), and the anti-inflammatory cytokine IL-10 (**d**) were measured by ELISA. **e** The ability of rapamycin to alleviate LPS-induced microglial activation was measured by staining Iba1 in dentate gyrus (DG) of hippocampus and cortex of mice. The white boxed areas are shown enlarged in the bottom left of each image. Graphs show the numbers of Iba1-positive microglia in DG (**f**) and cortex (**i**), the average process length of Iba1-positive microglia in DG (**g**) and cortex (**j**), and the number microglial endpoints in DG (**h**) and cortex (**k**). Data are presented as mean ± SEM. ^*^p < 0.05, ^**^p < 0.01, ^***^p < 0.001 vs sham; ^#^*p* < 0.05, ^##^*p* < 0.01, ^###^*p* < 0.001 vs LPS. Scale bar: 100 μm

To better evaluate the role of microglial autophagy in regulating LPS-induced neuroinflammation, we used ELISA and immunohistochemistry to examine microglial activation after LPS exposure in vivo. Mice were injected with rapamycin (0, 0.25, 0.5, 1 nmol for each mouse) 15 min before LPS administration (5 μg, i.c.v.). Brain tissues were examined 24 h later. The results showed that the injection of LPS dramatically increased the secretion of pro-inflammatory TNF-α, IL-β, and IL-6 (Fig. 6a–c) and reduced the levels of the anti-inflammatory factor IL-10 (Fig. 6d) in mouse hippocampus. Compared with LPS treatment alone, cotreatment with LPS and rapamycin significantly attenuated the secretion of pro-inflammatory factors and significantly increased the levels of IL-10, thus showing an obvious anti-inflammatory effect. The mRNA levels of the pro-inflammatory cytokines TNF-α, IL-1β, IL-6, and iNOS in the cortex were measured by qRT-PCR (Additional file 4: Figure S4a-d), which also showed that autophagy activated by rapamycin played an anti-inflammatory role. Next, we injected (i.c.v.) LPS with rapamycin (1 nmol) to explore the degree of microglial activation in hippocampus and cerebral cortex. The number of Iba1-positive microglia was significantly increased in hippocampus and cortex sections from mice injected with LPS (Fig. 6f, i). The normal ramified microglia were evident in the sham group, but fully activated and hypertrophic microglia were present 24 h after treatment with LPS, as judged by the lengths of the microglial processes and the number of microglial endpoints (Fig. 6e, g–h, j–k). The effect of LPS was reversed by rapamycin. These in vivo data confirmed that activation of autophagy can inhibit the production of pro-inflammatory cytokines caused by LPS and reduce the activation of microglia in hippocampus and cortex.

## Discussion

**Fig. 7:**
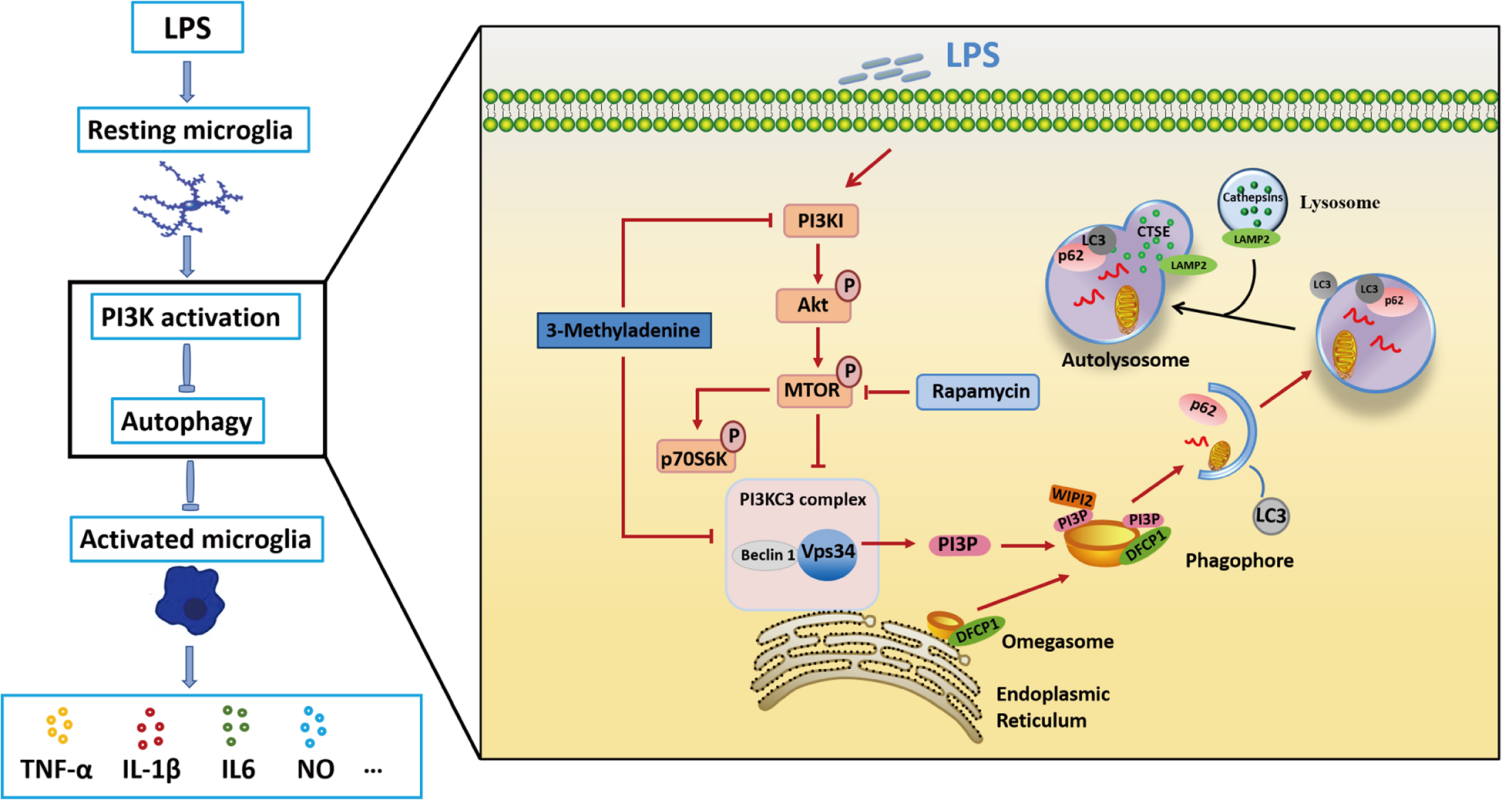
Schematic description of LPS-mediated suppression of autophagy, which leads to microglial activation. LPS activates MTOR via phosphorylation, leading to the decreased expression of Vps34 which causes a reduction of PI(3)P. The PI(3)P deficiency restricts maturation of omegasomes to phagophores and thus inhibits the formation of autophagosomes, which ultimately increases the activation of microglial cells

Our findings identified a key role of autophagy in influencing LPS-induced neuroinflammation. We showed that autophagy flux was impaired in LPS-stimulated N9 microglia. The impairment of autophagic flux in microglia is likely attributable to inhibition of autophagosome formation rather than disruption of the function of the lysosomal system. The inhibition of autophagy was caused by the downregulation of Vps34, which is involved in autophagosome biogenesis. To confirm this finding, we stably overexpressed Vps34 in N9 microglial cells by lentivirus infection. As expected, the data showed that overexpression of Vps34 was effective in combating the LPS-induced reduction in autophagy flux and reversing the LPS-induced accumulation of substrates. Moreover, rescue of autophagy by rapamycin, an autophagy inducer, significantly alleviated the LPS-induced neuroinflammation and restored the activation of microglia in mice. Furthermore, we found that overexpression of Vps34 also exerted a beneficial effect on the LPS-induced neuroinflammatory reaction (Fig. 7). To our knowledge, this is the first demonstration that Vps34 is involved in the regulation of autophagy in LPS-induced neuroinflammation. These results indicate that activation of microglial autophagy may be a potential target for alleviating neuroinflammation. Although it has been reported that LPS upregulates autophagy via the receptor TLR4 in RAW264.7 cells [46], Lee et al. and He et al. observed that autophagy was inhibited in LPS-induced BV2 microglia, which is consistent with our results [33, 47]. The study of Lee et al. and He et al. also examined the effect of LPS on autophagy in RAW264.7 cells and revealed a differential LC3 response to LPS between microglial BV2 cells, in which autophagy was downregulated, and RAW264.7 cells, in which autophagy was upregulated [47]. The researchers speculated that the mechanism of autophagy inhibition may be dependent on cell type or even the state of cells of the same type.

It is well known that autophagy works as a housekeeper in cells, similar to the role played by microglia in the CNS [20]. Accordingly, autophagy is thought to be essential for normal degradation and turnover of cellular constituents or debris [48]. It had been reported that lack of basal autophagy in the CNS leads to various neurodegenerative diseases, even in the absence of any disease-associated mutant protein [49–51]. Some reports revealed that inflammation is considerably limited by efficient autophagic responses in glial cells that survive stroke [52]. Moreover, in BV2 cells with knockdown of Atg5 to prevent autophagy, LPS promoted a further increase in the level of mature IL-1β [53]. In addition, there was an increased inflammation reaction following the disruption of microglial autophagy [48]. However, some researchers came up with the opposite view that the production of inflammatory mediators was caused by autophagy activation in microglial cells [8] or glial cells [54]. Until now, no definitive conclusion has been drawn on this issue.

To study whether the impairment of autophagic flux was caused by the inhibition of autophagosome formation or the dysfunction of lysosomes, we analyzed the expression of lysosome-related proteins and enzymes, and we used chloroquine to block downstream autophagosome/lysosome fusion. The results showed that lysosomal function was not changed by LPS, and LPS could still reduce the level of LC3-II and further increase the accumulation of SQSTM1 after CQ treatment. This suggests that the effect of LPS on autophagic flux might not be related with lysosomal dysfunction but rather with impairment of an early step in autophagosome synthesis. It is well known that in mammals the original site of autophagosome biogenesis is the omegasome [55], which forms at sites on the ER. Recruitment of Vps34 facilitates the maturation of omegasomes into phagophores. Phosphatidylinositol 3-phosphate (PtdIns3P, PI(3)P), generated by Vps34, is enriched in the inner lumen of the nascent autophagosome and serves as an anchor for recruitment of PI(3)P-binding proteins such as DFCP1 and WIPI2, which play a role in the formation of the early autophagosomal membrane. Here, we found that the expression of Vps34 was significantly inhibited in LPS-stimulated microglia. These results are consistent with a recent metabolomics analysis in microglia which showed that LPS induced a reduction in the synthesis of PI(3)P [47]. Therefore, it appears that LPS impairs autophagic flux in microglia by reducing the formation of autophagosomes. In previous studies, evidence indicated a selective deficiency in PI(3)P in the brain of AD patients and AD model mice. The researchers believed that the PI(3)P deficiency may reflect changes occurring in glial cells, since the deficiency was independent of the extent of neuronal death or AD pathology in AD-affected regions [56]. Microglial cells are now considered to be active participants in brain function and dysfunction. They surround amyloid plaques in human AD brains, and clear cellular debris and toxic proteins [57]. Our findings are important for understanding the mechanism by which microglial inflammation disrupts autophagy. In the case of LPS-induced inflammation, the inhibition of autophagic flux may be associated with a deficiency of autophagosome formation caused by a reduced level of Vps34 protein, which results in insufficient PI(3)P for the early steps of autophagosome biogenesis.

Recently, persuasive evidence has emerged to support the role of autophagy impairment in the pathogenesis of several major neurodegenerative diseases, and this evidence suggests therapeutic strategies that enhance autophagic flux by targeting the stages that are specifically disrupted in each disease [29, 58]. To further verify the relationship between the inhibition of autophagy and neuroinflammation, we used rapamycin, which inhibits the MTOR pathway, to observe its effect on the inflammatory response induced by LPS. We found that induction of autophagy by LPS in N9 microglial cells was associated with a remarkable reduction of pro-inflammatory factors, including NO, IL-6, and TNF-α. In our in vivo studies, we further demonstrated that LPS-induced neuroinflammation in the cortex and hippocampus, which are known to be involved in memory and cognition. Consistent with our in vitro studies, rapamycin significantly reduced the release of inflammatory cytokines and the activation of microglia in mouse brain. Taken together, our results provide evidence that rapamycin-induced activation of autophagy plays a critical role in inhibiting microglial activation and the release of pro-inflammatory cytokines.

Although we found that promotion of autophagy was able to alleviate neuroinflammation, our findings did not answer the question of how autophagy inhibits neuroinflammatory processes. It has been reported that a wide range of stimuli can activate the NLRP3 inflammasome signaling pathway, which is involved in triggering autophagy-lysosomal dysfunction, and increase the release of proinflammatory factors in microglial cells [53]. Dupont et al. demonstrated that autophagy is involved in mediating unconventional secretion of IL-1β [59–61]. In contrast, He et al. proposed that the inflammasome itself might be one of the likely targets that is negatively controlled by autophagy [33]. The mechanism by which autophagy regulates IL-1β release is obviously complicated and requires further investigation. We found that when we removed the culture medium and replaced it with fresh medium containing 1 μg/mL LPS every 6 h for 24 h, the inhibition of autophagy was significantly reduced (data not shown). Nevertheless, the molecular mechanisms underlying the induction of autophagy and neuroinflammation need further exploration.

## Conclusion

In summary, the present study reveals the role of microglial autophagy and the Vps34-related molecular mechanism in regulating LPS-induced neuroinflammation. These results provide evidence to support the idea that microglial autophagy represents a potential target for alleviating neurodegenerative diseases.

## Supplementary information

**Additional file 1: Figure S1.** LPS activates inflammation in microglial cells. N9 microglial cells were stimulated with LPS for 24 h in a dose-dependent manner (0-10 μg/mL). (a) Cell viability was measured using the MTT assay. (b) Culture supernatants were isolated and measured for NO production using Griess reagents. (c) N9 microglial cells were treated with 1 μg/mL LPS for the indicated times (0-24 h). Cell lysates were prepared, and protein levels of iNOS were analyzed with western blotting. (d) Quantification of (c). Data are presented as mean ± SEM. ^*^p < 0.05, ^**^p < 0.01, ^***^p < 0.001 vs control.

**Additional file 2: Figure S2.** Overexpression of Vps34 in N9 microglial cells. (a) The expression of endogenous and exogenous Vps34. WT, untreated N9 cells; OE, N9 cells transduced with lentivirus expressing Vps34; Vec, N9 cells transduced with lentivirus carrying empty vector. (b) The mRNA levels of the pro-inflammatory cytokines *TNF-α*, *IL-6* and *iNOS* in Vps34 overexpressing N9 microglial cells after treatment with 1 μg/mL LPS for 6 h were measured by qRT-PCR. Data are presented as mean ± SEM. ^$$^*p* < 0.01 vs wild type; ^#^*p* < 0.05, ^##^ *p* < 0.01 vs vector.

**Additional file 3: Figure S3.** (a) Representative TEM images of an N9 microglial cell. (b) Representative TEM images of an N9 microglial cell after treatment LPS for 12 h. (c) Representative TEM images of autophagosomes in an N9 microglial cell after treatment with rapamycin for 12 h. Boxed regions are shown enlarged in the adjacent panels. Scale bar: 500 nm (white), 1 μm (black). AP, autophagosome; ER, endoplasmic reticulum; EE, early endosome; LE, late endosome; Ly, lysosome; Mt, mitochondria; Nu, nucleus.

**Additional file 4: Figure S4.** Rapamycin alleviates neuroinflammation *in vivo* by activating autophagy. Different doses of rapamycin (0.25, 0.5, 1 nmol for each mouse) were administered via intracerebroventricular injection 15 min before 5 μg LPS. The mRNA levels of the pro-inflammatory cytokines *TNF-α* (a), *IL-1β* (b), *IL-6* (c) and *iNOS* (d) in the cortex were measured by qRT-PCR. Data are presented as mean ± SEM. ^*^p < 0.05, ^**^p < 0.01, ^***^p < 0.001 vs sham; ^#^*p* < 0.05, ^##^*p* < 0.01, ^###^*p* < 0.001 vs LPS.

## Data Availability

All the necessary data are included in the article. Further data will be shared by request.

## Abbreviations

239FT: Human embryonic kidney cells
3-MA: 3-Methyladenine
ACSF: Artificial cerebrospinal fluid
ANOVA: Analysis of variance
ATG5: Autophagy-related protein 5
Baf A1: Bafilomycin A1
CM: Conditioned medium
CNS: Central nervous system
CQ: Chloroquine
CSTE: Cathepsin E
DAB: 3,3′-diaminobenzidine
DFCP1: Double FYVE–containing protein 1
DMEM: Dulbecco’s modified Eagle’s medium
ELISA: Enzyme-linked immunosorbent assay
ER: Endoplasmic reticulum
FBS: Fetal bovine serum
GFP: Green fluorescent protein
i.c.v.: Intracerebroventricular injection
i.p.: Intraperitoneal injection
Iba1: Ionized calcium–binding adaptor molecule-1
IL-10: Interleukin-10
IL-1β: Interleukin-1β
IL-6: Interleukin-6
IMDM: Iscove’s modified Dulbecco’s medium
iNOS: Inducible nitric oxide synthase
LAMP2: Lysosomal-associated membrane protein 2
LPS: Lipopolysaccharide
MAP1LC3/LC3: Microtubule-associated protein 1 light chain 3
Mat-: Mature form
MTOR: Mechanistic target of rapamycin
MTT: 3-[4,5-dimethylthiazol-2-yl]-2,5-diphenyltetrazolium bromide
NO: Nitric oxide
NO_2_^−^: Nitrite
OTC: Optimal cutting temperature compound
p70S6K: Ribosomal protein s6 kinase
PFA: Paraformaldehyde
PI(3)P: Phosphatidylinositol 3-phosphate
PI3K: Phosphoinositide 3-kinase
PKB: Protein kinase B
Pro-: Precursor form
RFP: Red fluorescent protein
ROS: Reactive oxygen species
RT-PCR: Reverse transcription polymerase chain reaction
SH-SY5Y: Human neuroblastoma cells
SQSTM1: Sequestosome-1
TEM: Transmission electron microscopy
TNF-α: Tumor necrosis factor α
Vps34: Vacuolar protein sorting 34
WIPI2: WD-repeat domain, phosphoinositide interacting
