# Supplementary figures and images for: Lipopolysaccharide induces neuroinflammation in microglia by activating the MTOR pathway and downregulating Vps34 to inhibit autophagosome formation
Source: J Neuroinflammation. 2020 Jan 11;17:18. doi: 10.1186/s12974-019-1644-8 (PMC6954631; doi:10.1186/s12974-019-1644-8)

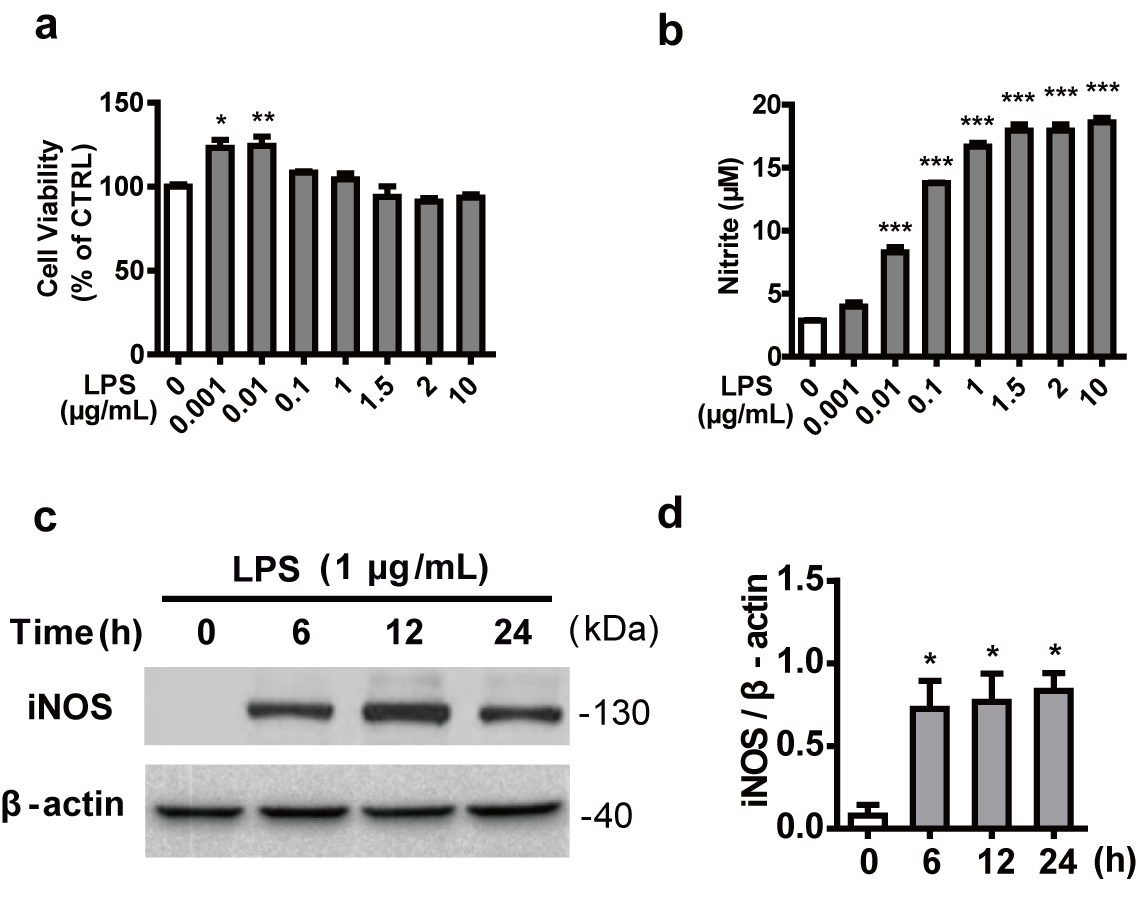

Supplement: Supplementary file 1 — Additional file 1: Figure S1. LPS activates inflammation in microglial cells. N9 microglial cells were stimulated with LPS for 24 h in a dose-dependent manner (0-10 μg/mL). (a) Cell viability was measured using the MTT assay. (b) Culture supernatants were isolated and measured for NO production using Griess reagents. (c) N9 microglial cells were treated with 1 μg/mL LPS for the indicated times (0-24 h). Cell lysates were prepared, and protein levels of iNOS were analyzed with western blotting. (d) Quantification of (c). Data are presented as mean ± SEM. *p < 0.05, **p < 0.01, ***p < 0.001 vs control. [file 12974_2019_1644_MOESM1_ESM.tif]

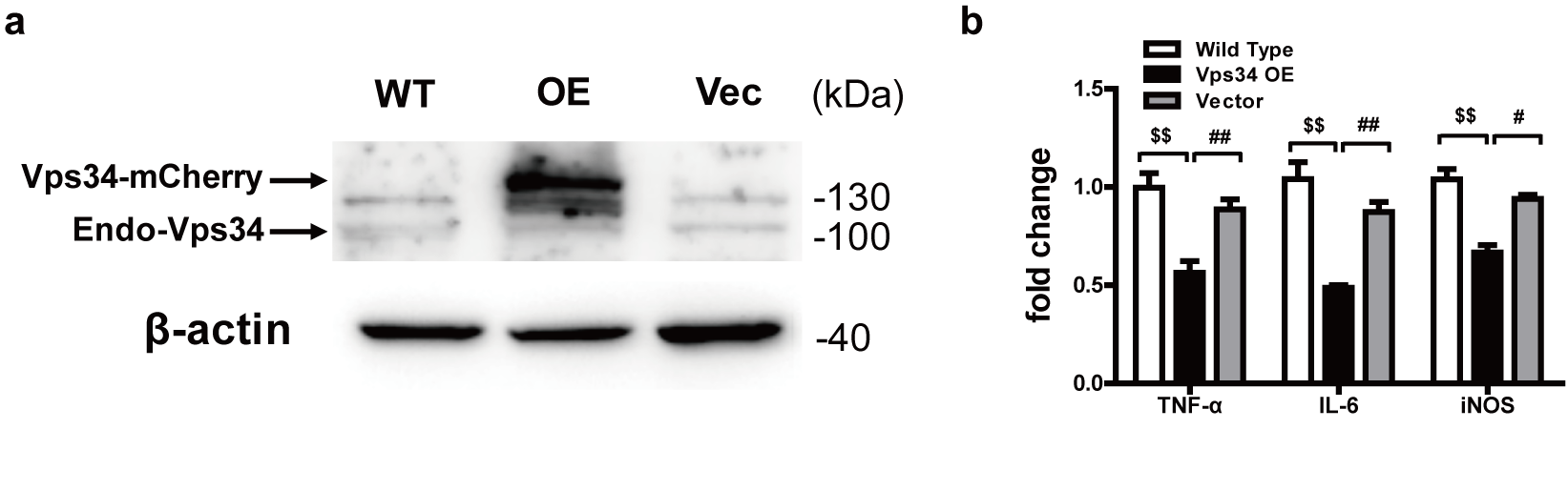

Supplement: Supplementary file 2 — Additional file 2: Figure S2. Overexpression of Vps34 in N9 microglial cells. (a) The expression of endogenous and exogenous Vps34. WT, untreated N9 cells; OE, N9 cells transduced with lentivirus expressing Vps34; Vec, N9 cells transduced with lentivirus carrying empty vector. (b) The mRNA levels of the pro-inflammatory cytokines TNF-α, IL-6 and iNOS in Vps34 overexpressing N9 microglial cells after treatment with 1 μg/mL LPS for 6 h were measured by qRT-PCR. Data are presented as mean ± SEM. $$p < 0.01 vs wild type; #p < 0.05, ## p < 0.01 vs vector. [file 12974_2019_1644_MOESM2_ESM.tif]

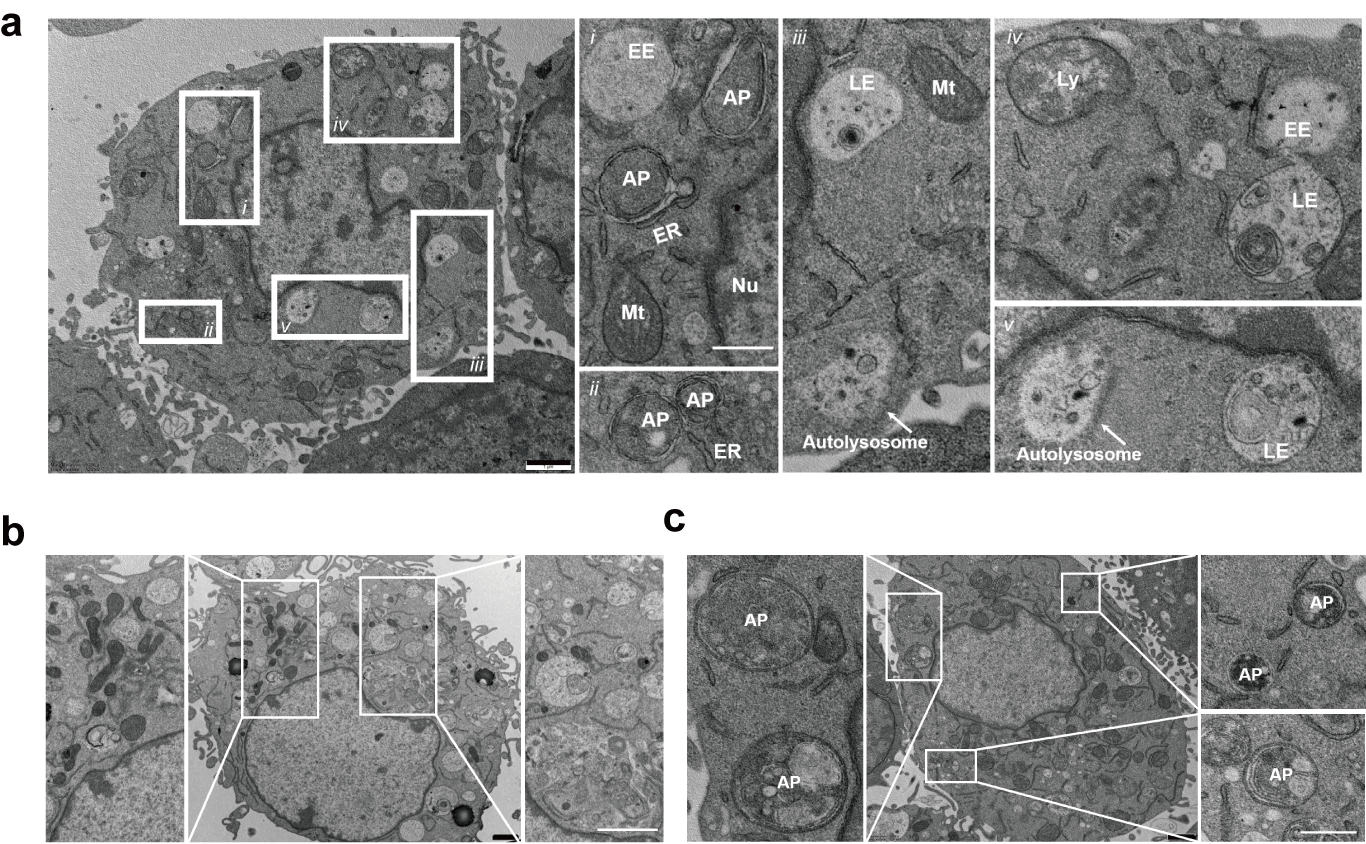

Supplement: Supplementary file 3 — Additional file 3: Figure S3. (a) Representative TEM images of an N9 microglial cell. (b) Representative TEM images of an N9 microglial cell after treatment LPS for 12 h. (c) Representative TEM images of autophagosomes in an N9 microglial cell after treatment with rapamycin for 12 h. Boxed regions are shown enlarged in the adjacent panels. Scale bar: 500 nm (white), 1 μm (black). AP, autophagosome; ER, endoplasmic reticulum; EE, early endosome; LE, late endosome; Ly, lysosome; Mt, mitochondria; Nu, nucleus. [file 12974_2019_1644_MOESM3_ESM.tif]

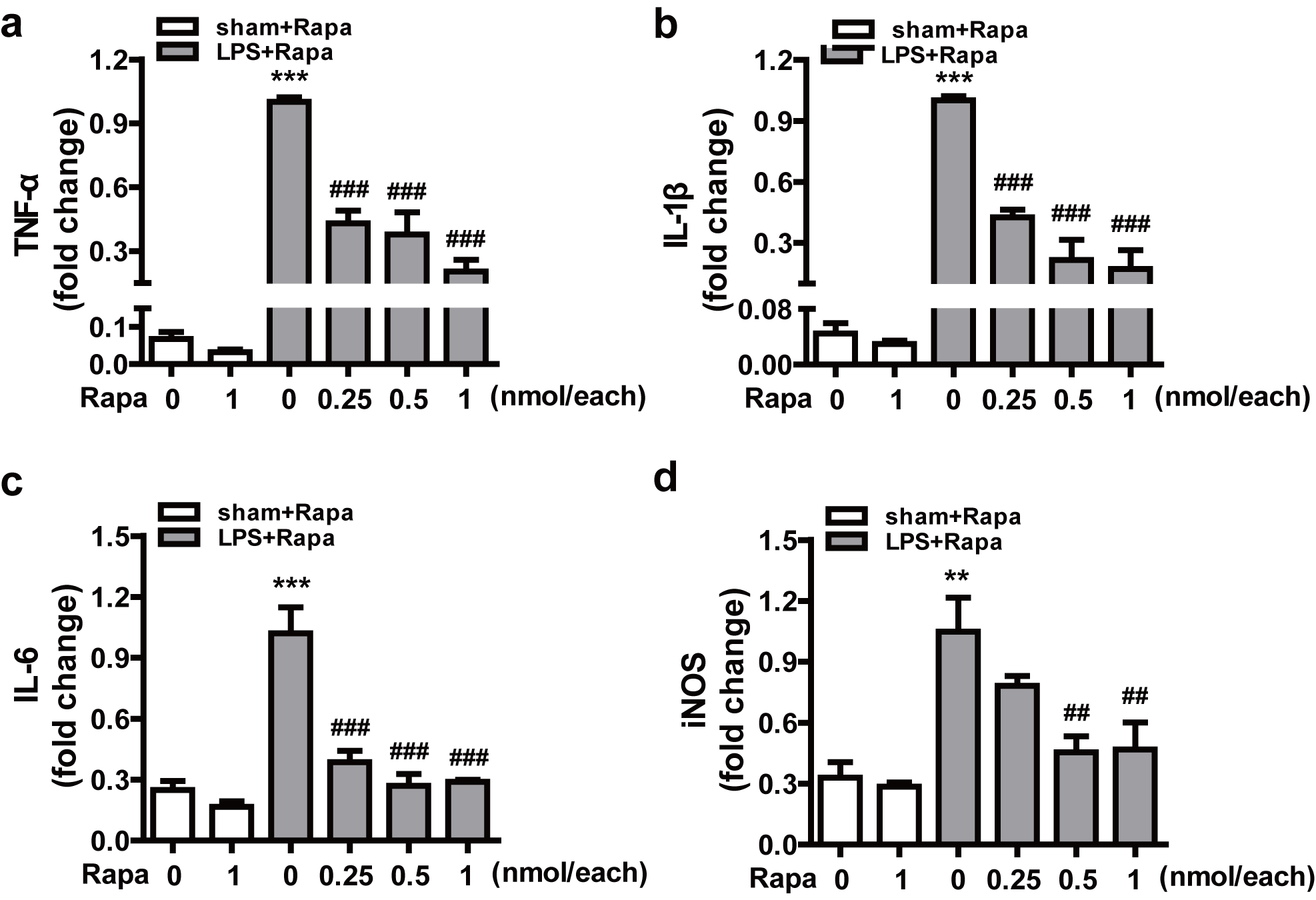

Supplement: Supplementary file 4 — Additional file 4: Figure S4. Rapamycin alleviates neuroinflammation in vivo by activating autophagy. Different doses of rapamycin (0.25, 0.5, 1 nmol for each mouse) were administered via intracerebroventricular injection 15 min before 5 μg LPS. The mRNA levels of the pro-inflammatory cytokines TNF-α (a), IL-1β (b), IL-6 (c) and iNOS (d) in the cortex were measured by qRT-PCR. Data are presented as mean ± SEM. *p < 0.05, **p < 0.01, ***p < 0.001 vs sham; #p < 0.05, ##p < 0.01, ###p < 0.001 vs LPS. [file 12974_2019_1644_MOESM4_ESM.tif]
